# Comparative assessment of pulse transit time–derived blood pressure and ambulatory blood pressure monitoring in patients with obstructive sleep apnea

**DOI:** 10.1007/s11325-026-03601-6

**Published:** 2026-02-11

**Authors:** Sathida Traiwannakij, Athiwat Tripipitsiriwat, Sirisawat Kunanon, Duangporn Lertsilp, Pakanach Promkeam-on, Pakpoom Choeycheep, Nitipatana Chierakul

**Affiliations:** 1 Department of Medicine, Faculty of Medicine Siriraj Hospital, Mahidol University, Bangkok, Thailand; 2 Division of Respiratory Disease and Tuberculosis, Department of Medicine, Faculty of Medicine Siriraj Hospital, Mahidol University, Bangkok, Thailand; 3 Division of Hypertension, Department of Medicine, Faculty of Medicine Siriraj Hospital, Mahidol University, Bangkok, Thailand; 4 Siriraj Sleep Center, Faculty of Medicine Siriraj Hospital, Mahidol University, Bangkok, Thailand

**Keywords:** Continuous blood pressure monitoring, Cuffless blood pressure monitoring, Diagnostic accuracy, Nocturnal blood pressure, Sleep-disordered breathing, Sleep test

## Abstract

**Study objectives:**

Nocturnal hypertension is common in patients with obstructive sleep apnea (OSA) and contributes to elevated cardiovascular risk. Pulse transit time (PTT) offers a noninvasive method to estimate nocturnal blood pressure (BP) during sleep, but its clinical validity in OSA remains unclear. This study aimed to assess the correlation between PTT-derived nocturnal BP (PTT-BP) and 24-hour ambulatory BP monitoring (ABPM-BP) in patients with suspected OSA.

**Methods:**

Adults undergoing full-night PSG for suspected OSA were prospectively enrolled and underwent 24-hour ABPM the following day. Nocturnal hypertension was defined as mean nighttime BP ≥ 120/70 mmHg. Exclusion criteria included positive airway pressure titration, arrhythmias affecting PTT accuracy, and incomplete ABPM data. PTT-BP and ABPM-BP were compared across OSA severity levels.

**Results:**

Sixty-six subjects (median age 47 years; 39.4% male) were analyzed. Prior hypertension was reported in 40.9%. OSA severity was mild in 28.8%, moderate in 37.9%, and severe in 28.8%. PTT-BP demonstrated a weak correlation with nocturnal ABPM-BP. Among patients with mild OSA, nocturnal PTT- BP was comparable to ABPM-BP. However, among patients with moderate to severe OSA, both systolic and diastolic PTT-BP exceeded ABPM-BP. Sensitivity and specificity of PTT-SBP ≥ 104 mmHg in detecting nocturnal hypertension were 85% and 39%, respectively. PTT-SBP ≥ 104 mmHg identified masked hypertension with 78% sensitivity and 48% specificity.

**Conclusions:**

PTT-derived BP tends to overestimate nocturnal BP in patients with moderate to severe OSA. The exact value from PTT can be significantly different from ambulatory blood pressure monitoring, which affects reliability. However, PTT-derived systolic BP may be useful in screening for nocturnal and masked hypertension.

**Supplementary Information:**

The online version contains supplementary material available at 10.1007/s11325-026-03601-6.

## Introduction

Obstructive sleep apnea (OSA) is a sleep-related breathing disorder marked by intermittent collapse of the upper airway, leading to reduced or halted airflow despite intact respiratory effort [1]. Multiple pathophysiological mechanisms in OSA could elevate blood pressure (BP) and can result in chronic hypertension, placing these patients at higher risk for cardiovascular comorbidities [2]. Effective BP management is therefore critical to mitigate adverse cardiovascular outcomes.

Assessment of BP is recommended for patients with OSA [3], yet no official guidelines address optimal methods for monitoring nighttime BP. Abnormal respiratory events predominantly occur during sleep, and nocturnal hypertension is common in this population [4]. Individuals with nocturnal hypertension are more prone to cardiac and carotid structural changes than those without nocturnal hypertension [5]. Even isolated nocturnal hypertension elevates the risk of adverse cardiovascular outcomes [6]. Numerous studies have shown that nighttime BP and dipping status are strong predictors of adverse events, including cardiovascular events and mortality [7, 8].

At present, ambulatory BP monitoring (ABPM) remains the standard for diagnosing nocturnal hypertension, defined as a nocturnal systolic/diastolic BP of ≥ 120/70 mmHg [9]. However, ABPM relies on a cuff-based oscillometric method that cannot provide continuous measurements during sleep. Each measurement offers only a discrete value, which may fail to capture transient BP surges related to respiratory events [10, 11]. ABPM measurements can also disrupt sleep, thereby reducing reliability and tolerance in some patients [12].

An intriguing alternative involves estimating nocturnal BP using physiological signals recorded during polysomnography (PSG). Pulse transit time (PTT) is derived from electrocardiogram and peripheral plethysmography signals, which are standard components of PSG [13]. Algorithms that convert PTT into BP readings have been developed and validated [13]. Nevertheless, limited data compare PTT-derived BP with ABPM in clinical settings, and these studies report only moderate agreement [14, 15]. Some findings indicate that PTT-derived BP may overestimate ABPM values, especially at night, but the role of sleep-disordered breathing in this discrepancy is unclear. A few investigations have examined PTT-derived BP during PSG in patients with sleep disorders, these results suggest potential feasibility [16]. However, the higher prevalence of increased arterial stiffness in patients with OSA might impact pulse wave velocity (PWV), potentially compromising PTT-based algorithms that rely on assumptions of standard arterial elasticity [2, 14]. Consequently, further research focusing on this population is warranted.

In this study, we evaluated the correlation between nocturnal hypertension identified by ABPM and PTT-derived BP in patients referred for overnight PSG due to suspected OSA. Our findings may help identify OSA patients at risk for nocturnal hypertension through a more accessible, continuous, and potentially less disruptive method of BP measurement.

## Methods

This study was conducted prospectively at the Sleep Center of the Faculty of Medicine Siriraj Hospital, Mahidol University, a tertiary care hospital, from February 1, 2024, to September 30, 2024. The institutional review board of Mahidol University approved the protocol and provided oversight (Si 482/2023).

### Study population

Adult patients older than 18 years with suspected OSA who were referred for overnight PSG were screened consecutively. Eligible participants underwent 24-hour ABPM the day after their PSG. Patients were excluded if they received positive airway pressure titration (split-night testing), had conditions that might compromise PTT reliability (such as atrial fibrillation), or had incomplete or intolerable ABPM recordings.

### PSG and scoring

PSG was performed using the SOMNO HD EEG 32 system (SOMNOmedics AG, Randersacker, Germany). The recording montage included electroencephalography, chin electromyography, electrooculography, electrocardiography, bilateral leg electromyographies, oxygen saturation (SpO₂), nasal transducer, thermistor, chest and abdominal respiratory inductance plethysmography, and PTT-derived BPP measured by SOMNOtouch NIBP system (SOMNOmedics AG). Certified sleep technologists conducted the monitoring and scoring according to guidelines endorsed by the American Academy of Sleep Medicine [17].

Respiratory events were defined as follows. Obstructive apnea was a reduction in airflow of more than 90% of baseline, along with continued respiratory effort for longer than 10 s. Hypopnea was a reduction in airflow exceeding 30% of baseline for longer than 10 s, accompanied by either a 3% oxygen desaturation or an arousal. The apnea-hypopnea index was calculated as the total number of apneas plus hypopneas per hour of sleep. OSA severity was categorized by the apnea-hypopnea index as mild (5 to < 15 events/hour), moderate (15 to < 30 events/hour), and severe (≥ 30 events/hour).

### PTT and PTT-derived BP

PTT is the time interval required for a pulse pressure wave to travel from the left ventricle to the fingertip [18]. The R-peak of the electrocardiographic signal represents left-ventricular ejection, while the turning point of the finger pulse waveform in the plethysmography signal denotes wave arrival [18]. PTT correlates closely with pulse wave velocity (PWV) and BP.

The SOMNOtouch NIBP system (SOMNOmedics AG) meets the validation criteria of the European Society of Hypertension International Protocol and provides continuous, cuffless 24-hour BP monitoring [13]. This device computes PTT using plethysmographic signals captured by a finger probe during PSG. The same finger probe is also used to measure peripheral oxygen saturation.

Before collecting PTT-based BP readings, each patient received a conventional cuff-BP measurement for calibration. A single-point calibration was then used to align PTT, BP, and vascular properties. By incorporating the patient’s height—an estimate of pulse wave path length—this algorithm computes systolic BP (SBP) and diastolic BP (DBP) values for each PTT measurement. The relationship between PWV, height, body correlation factor (BDC) and PTT are illustrated as follows [18]:

$$\:PWV\left(cm/ms\right)=BDC\:x\frac{height\left(cm\right)}{PTT\left(ms\right)}$$

A proprietary algorithm within the DOMINO software (version 3.0.0.8) refines these calculations. During PSG, the system converts each PTT value into a beat-by-beat BP measurement. Mean SBP and DBP are derived from the total recording time. The lowest SBP and DBP are computed by averaging 10-minute intervals of PTT-derived BP centered around the respective nadirs.

### 24-hour ABPM

Twenty-four-hour ABPM was performed using WatchBP 03 (Microlife WatchBP AG, Widnau, Switzerland), a clinically validated device meeting European Society of Hypertension and ISO 81,060‒2:2013 standards. This apparatus measures SBP, DBP, mean arterial pressure, and pulse rate through a noninvasive oscillometric technique with an inflatable upper-arm cuff.

A valid ABPM recording was defined as having at least 20 BP readings during wakefulness and 7 readings during sleep. ABPM intolerance was characterized by an inability to continue monitoring due to adverse reactions, including skin rash, pruritus at the cuff site, or arm swelling.

Daytime was defined as the period from self-reported morning wake time until bedtime, whereas nighttime was defined as the period from bedtime until morning wake time. Participants were instructed to adhere to their usual daily activities and maintain a sleep-wake schedule consistent with the previous night’s PSG study.

### Collected data and outcome measures

Demographic data, anthropometric measurements, hypertension status (including antihypertensive medication use), and comorbidities were collected via interviews, physical examinations, and reviews of medical records. A hypertension diagnosis was defined as one previously confirmed by a physician. Sleep parameters were obtained from PSG. PTT-derived BP, including mean systolic and diastolic values, was extracted from raw data. In this study, nighttime PTT-derived BP refers to the average value recorded by the PSG device. ABPM-derived BP, including mean daytime and mean nighttime readings and dipping status, was obtained by reviewing 24-hour ABPM recordings.

The clinical criteria of hypertension by ABPM were determined using the 2023 European Society of Hypertension guidelines: mean daytime BP ≥ 135/85 mmHg or mean nighttime BP ≥ 120/70 mmHg [19]. Nocturnal hypertension derived from PTT and ABPM were defined using the same cutoff (mean nighttime BP ≥ 120/70 mmHg), but these were analyzed and reported separately (PTT-derived nocturnal hypertension vs. ABPM-derived nocturnal hypertension). Elevation of mean daytime BP by ABPM in patients without a history of hypertension would also be reviewed. Individuals without a history of hypertension and normal office BP who exhibited elevated mean BP by ABPM, either daytime or nighttime, were considered to have masked hypertension.

Dipping pattern was defined by the ratio of mean nighttime ABPM-derived BP to mean daytime ABPM-derived BP. Extreme dipper was < 0.8, dipper was 0.8‒0.9, non-dipper was > 0.9‒1.0, and reverse dipper was > 1.0.

### Statistical analysis

Baseline characteristics were reported as frequencies (percentages) for categorical variables and medians with interquartile ranges for continuous variables. The Wilcoxon signed-rank test compared ABPM- and PTT-derived BP. Spearman’s rank correlation assessed the relationship between these two methods. The Kruskal‒Wallis test evaluated differences in BP among OSA severity groups; if significant, pairwise comparisons followed using the Wilcoxon rank-sum test with Bonferroni correction.

The diagnostic performance of PTT-derived BP for detecting nocturnal hypertension and masked hypertension was evaluated based on sensitivity, specificity, positive predictive value, and negative predictive value. The area under the receiver operating characteristic curve was also determined. Youden’s index (sensitivity + specificity – 1) was calculated for each BP threshold. The cutoff value yielding the highest Youden’s index was identified as the optimal cutoff. This analysis was restricted to patients with OSA, because patients without OSA may have other reasons for elevated nocturnal BP and masked hypertension.

Statistical significance was defined as a *p*-value less than 0.05. All analyses were performed using R (version 4.4.2; R Core Team, Vienna, Austria) and RStudio (version 2024.12; Posit Team, Boston, MA, USA).

## Results

**Fig. 1:**
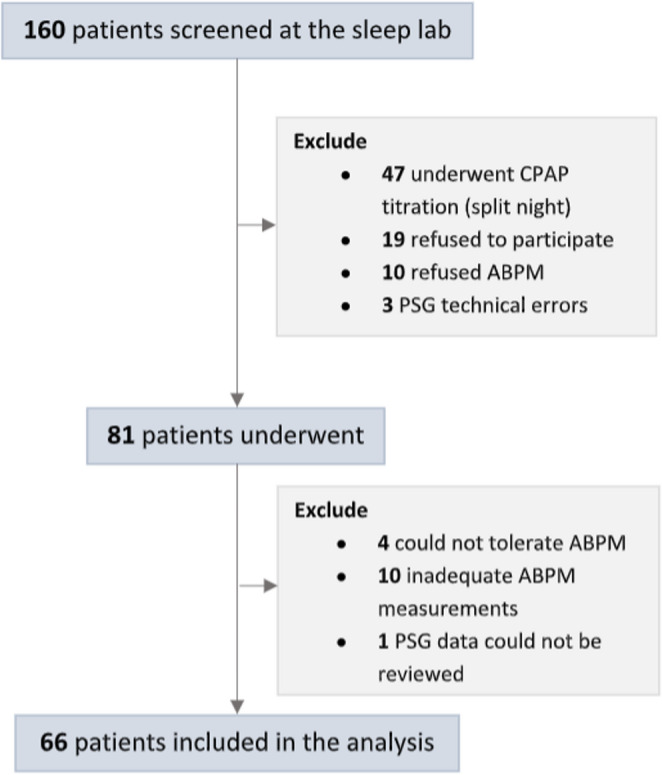
Enrollment flow diagram of the study cohort

A total of 160 patients were screened for eligibility during the enrollment period. The enrollment flow and reasons for exclusion are depicted in Fig. 1. Ultimately, 66 patients completed both PSG and ABPM, fulfilling the criteria for adequate testing. Baseline characteristics of these 66 patients are presented in Table 1. Before PSG, 40.9% of participants had physician-diagnosed hypertension, and 57.5% showed a non-dipping BP pattern (non-dippers or reverse dippers).

**Table 1:** Baseline characteristics of the analytic cohort (*n* = 66)*

Characteristics	*N* = 66 (median (IQR) or n(%))
Age (years)	47 (36, 60)
Male, n (%)	26/66 (39.4%)
BMI, kg/m^2^	28 (24.9, 32)
HT (physician-based diagnosis), n (%)	27/66 (40.9%)
OSA	
Mild, n (%)	19 (28.8%)
Moderate, n (%)	25 (37.9%)
Severe, n (%)	19 (28.8%)
No (AHI < 5 events/hour)	3 (4.5%)
Median AHI, events/hour	20.6 (11.9, 30.5)
ODI3%, events/hour	8.9 (5.3, 16.7)
SpO_2_ nadir, %	87 (82, 90)
Time SpO_2_ <90%, %TST	0.1 (0, 1.2)
Sleep architecture	
Sleep efficiency, %	79.5 (69.4, 87.5)
N1, %	10.4 (6.8, 20.3)
N2, %	53.7 (44.6, 60.3)
N3, %	14.4 (10.1, 22.5)
REM, %	17 (12.6, 21.6)
WASO, min	83 (52.8, 112)
PLMI, events/h	0.1 (0, 3)
PTT-derived BP	
SBP, mmHg	114 (102, 121)
DBP, mmHg	72 (65, 78)
ABPM-derived BP	
SBP (daytime), mmHg	117 (110, 124)
DBP (daytime), mmHg	73 (68, 78)
SBP (nighttime), mmHg	107 (99, 116)
DBP (nighttime), mmHg	65 (58, 70)
%dipping, %	7.4 (3, 12)
Extreme dipper, n (%)	3 (4.5%)
Dipper, n (%)	25 (37.9%)
Non-dipper, n (%)	29 (43.9%)
Reverse dipper, n (%)	9 (13.6%)

*Continuous variables are expressed as median (interquartile range); categorical variables are shown as counts and percentages

Abbreviations: *ABPM* 24-hour ambulatory blood pressure monitoring, *AHI* apnea-hypopnea index, *BMI* body mass index, *DBP* diastolic blood pressure, *HT* physician-diagnosed hypertension, *ODI* oxygen-desaturation index, *OSA* obstructive sleep apnea, *PLMI* periodic-limb-movement index, *PTT* pulse transit time, *SBP* systolic blood pressure, *WASO* wakefulness after sleep onset

**Fig. 2:**
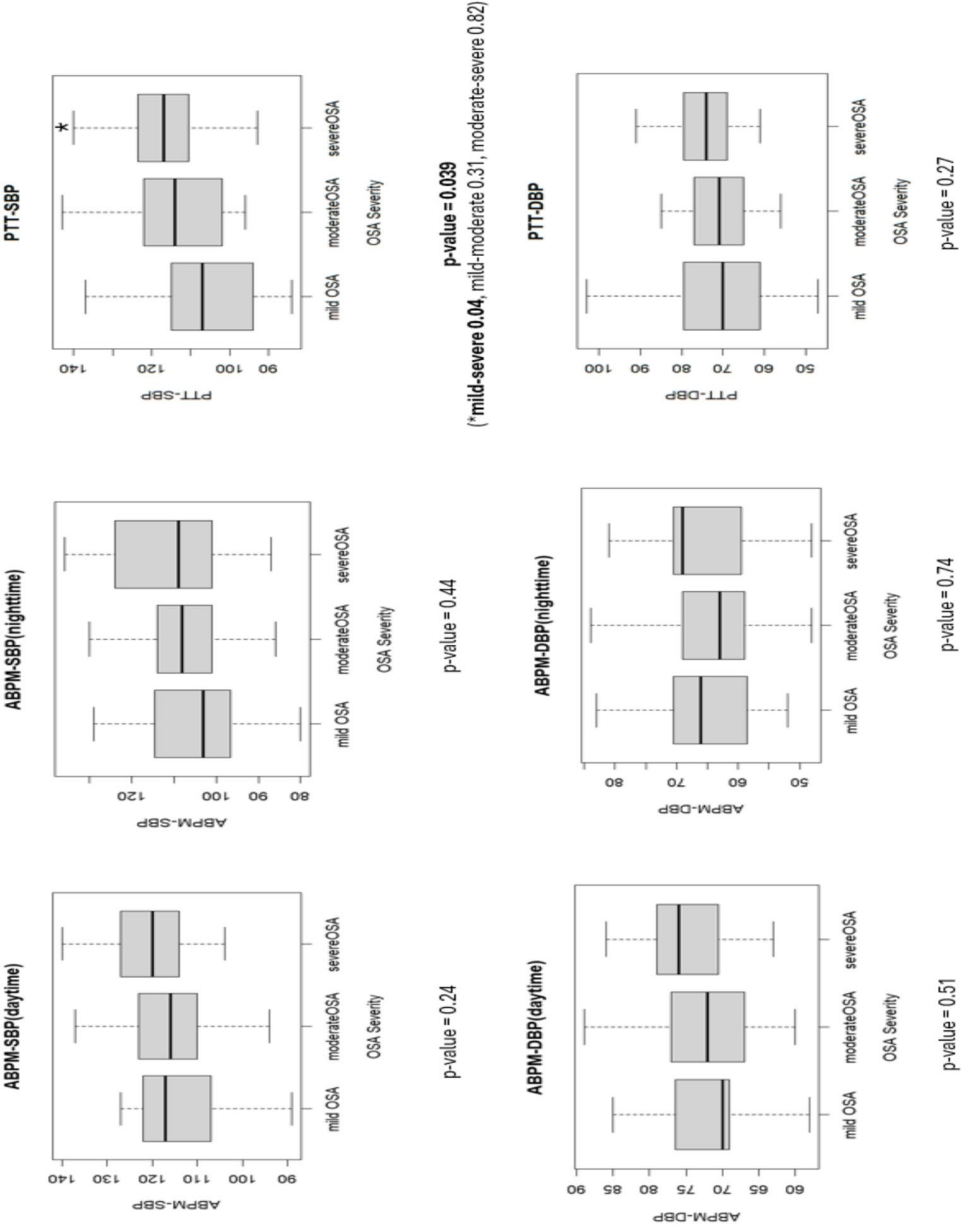
Ambulatory-derived versus pulse transit time–derived blood pressure across obstructive sleep apnea severity Box-and-whisker plots compare systolic and diastolic blood pressure (mm Hg) obtained by ambulatory monitoring and by pulse-transit-time analysis in mild, moderate, and severe obstructive sleep apnea. The central line represents the median; box edges mark the first and third quartiles; whiskers extend to 1.5 × the interquartile range

Among the 66 patients who completed both tests, 63 were diagnosed with OSA. Of these, 28.8% had mild OSA, 37.8% had moderate OSA, and 28.8% had severe OSA. Trends toward higher PTT- and ABPM-derived BP were observed with increasing OSA severity. However, statistically significant elevation was identified only for PTT-derived SBP in the severe OSA group compared to the mild OSA group (Fig. 2).

### PTT- versus ABPM-derived nighttime BP

A weak positive linear correlation was observed between PTT-derived SBP and ABPM-derived nighttime SBP (*r* = 0.25, *p* = 0.04), and between PTT-derived DBP and ABPM-derived nighttime DBP (*r* = 0.26, *p* = 0.03). Table 2 illustrates the comparison of nighttime BP measurements derived from PTT and ABPM. Overall, average PTT-derived nighttime SBP was similar to ABPM-derived nighttime SBP (114 mmHg vs. 107 mmHg; *p* = 0.09), with a mean difference of 4 mmHg (95% CI − 1 to 8). In contrast, PTT-derived nighttime DBP was significantly higher than ABPM-derived nighttime DBP (72 mmHg vs. 65 mmHg; *p* < 0.001), with a mean difference of 7 mmHg (95% CI 4 to 10).

**Table 2:** Nighttime blood pressure obtained by pulse-transit-time analysis versus ambulatory monitoring, overall and by obstructive sleep apnea severity*

	PTT-derived (median (IQR))	ABPM-derived(median (IQR))	*p*-value**	MD (95% CI)
All patients				
Avg nighttime SBP	114 (102, 121)	107 (99, 116)	0.09	4 (-1, 8)
Avg nighttime DBP	72 (65, 78)	65 (58, 70)	< 0.001	7 (4, 10)
Mild OSA				
Avg nighttime SBP	107 (94, 115)	103 (97, 115)	0.97	-1 (-9, 11)
Avg nighttime DBP	70 (61, 80)	66 (59, 71)	0.11	5 (-2, 14)
Moderate OSA				
Avg nighttime SBP	114 (102, 122)	108 (101, 114)	0.06	6 (-1, 12)
Avg nighttime DBP	71 (65, 77)	63 (59, 69)	0.014	8 (2, 11)
Severe OSA				
Avg nighttime SBP	117 (111, 124)	109 (101, 124)	0.33	6 (-5, 15)
Avg nighttime DBP	74 (69, 80)	69 (60, 71)	0.004	10 (3, 16)
Moderate/severe OSA				
Avg nighttime SBP	116 (105, 123)	109 (101, 116)	0.045	6 (0, 11)
Avg nighttime DBP	72 (67, 78)	65 (59, 70)	<0.001	8 (4, 12)

* Values are medians (interquartile ranges)

** *p*-values were calculated with the Wilcoxon sign-ranked test

Abbreviations: *ABPM* 24-hour ambulatory blood pressure monitoring, *DBP* diastolic blood pressure, *MD* mean paired difference, *OSA* obstructive sleep apnea, *PTT* pulse transit time, *SBP* systolic blood pressure, *IQR* interquartile range, *95%CI* 95% confidence interval

Within OSA severity subgroups, PTT-derived DBP was significantly higher than ABPM-derived nighttime DBP in both the moderate and severe OSA groups. No significant differences were seen in the mild OSA subgroup.

### Nocturnal hypertension

Using ABPM as the reference standard for nocturnal hypertension (mean nighttime BP ≥ 120/70 mmHg), 20 (31.7%) of patients with OSA met the criterion. PTT demonstrated 70% sensitivity and 37.2% specificity in detecting elevated nighttime BP (Table 3). The receiver operating characteristic curves for PTT-derived SBP and PTT-derived DBP showed areas under the curve (AUCs) of 0.62 (95% CI 0.48 ‒ 0.77) and 0.58 (95% CI 0.42 ‒ 0.74), respectively. Complete AUC coordinates for PTT-derived SBP and PTT-derived DBP can be found in the supplementary material (Figs. 3 and 4).

**Table 3:** Diagnostic performance of pulse-transit-time thresholds for detecting nocturnal hypertension (reference: ambulatory blood pressure monitoring nighttime mean ≥ 120/70 mm Hg)*

	PTT-BP ≥ 120/70	PTT-SBP ≥ 104	PTT-DBP ≥ 78
Sensitivity	0.70 (0.48–0.88)	0.85 (0.62–0.97)	0.40 (0.19–64.19)
Specificity	0.37 (0.23–0.53)	0.39 (0.25–0.56)	0.74 (0.59–86.59)
Positive predictive value	0.34 (0.20–0.51)	0.40 (0.25–0.56)	0.42 (0.20–0.67)
Negative predictive value	0.73 (0.50–0.89)	0.85 (0.62–0.97)	0.73 (0.57–0.85)
Accuracy	0.48 (0.35–0.61)	0.54 (0.41–0.67)	0.63 (0.50–0.75)

* Sensitivity, specificity, predictive values, and overall accuracy are presented with exact 95% confidence intervals

The column “PTT-BP ≥ 120/70” reflects the conventional hypertension cutoff; the PTT-SBP and PTT-DBP columns apply thresholds identified by Youden’s index (104 mm Hg and 78 mm Hg, respectively)

Abbreviations: *DBP* diastolic blood pressure (mmHg), *PTT* pulse transit time, *SBP* systolic blood pressure (mmHg)

Youden’s index (sensitivity + specificity ‒ 1) was used to explore alternative diagnostic thresholds for PTT-derived BP. The optimal cutoffs identified were 104 mmHg for SBP and 78.5 mmHg for DBP. Table 3 presents the diagnostic performance of these varying proposed PTT-derived BP thresholds for detecting nocturnal hypertension.

### Masked hypertension

Among the 36 patients with OSA who had no prior hypertension diagnosis, 9 (25%) showed abnormal mean BP from daytime or nighttime ABPM readings indicative of masked hypertension. Only one patient in this subgroup had abnormal daytime BP without elevated nighttime BP. The prevalence of masked hypertension in severe OSA was 50% (4 of 8), compared to 13.3% (2 of 15) in moderate OSA and 23.1% (3 of 13) in mild OSA.

Abnormal PTT-derived BP (≥ 120/70 mmHg) occurred in 24 of the 36 (66.67%) patients in this subgroup. Table 4 summarizes the diagnostic performance of PTT-derived BP for detecting masked hypertension, using the modified cutoffs derived via Youden’s index. The receiver operating characteristic curves for PTT-derived SBP and PTT-derived DBP showed AUC values of 0.58 (95% CI 0.38 ‒ 0.77) and 0.51 (95% CI 0.29 ‒ 0.73), respectively. Corresponding coordinates are provided in the supplementary material (Figs. 5 and 6).

**Table 4:** Diagnostic performance of pulse-transit-time thresholds for detecting masked hypertension in participants without prior hypertension (reference: abnormal daytime or nighttime ambulatory blood pressure monitoring)

	PTT-BP ≥ 120/70	PTT-SBP ≥ 104	PTT-DBP ≥ 64
Sensitivity	0.67 (0.30–0.93)	0.78 (0.40–0.97)	0.88 (0.52–99.52)
Specificity	0.33 (0.17–0.54)	0.48 (0.29–0.68)	0.26 (0.11–46.11)
Positive predictive value	0.25 (0.10–0.47)	0.33 (0.15–0.57)	0.29 (0.13–0.49)
Negative predictive value	0.75 (0.43–0.95)	0.87 (0.60–0.98)	0.88 (0.47–0.99)
Accuracy	0.42 (0.26–0.59)	0.56 (0.38–0.72)	0.42 (0.26–0.59)

* Metrics are reported as point estimates with exact 95% confidence intervals

The PTT-SBP (104 mm Hg) and PTT-DBP (64 mm Hg) thresholds were derived from Youden’s index in this subgroup

Abbreviations: *DBP* diastolic blood pressure (mmHg), *PTT* pulse transit time, *SBP* systolic blood pressure (mmHg)

## Discussion

We compared BP parameters derived from PTT during overnight PSG with those obtained from ABPM. Two principal findings were: (1) in patients with moderate and severe OSA, PTT-derived mean BP overestimated ABPM-derived values, and (2) elevated PTT-derived SBP may indicate nocturnal hypertension and masked hypertension.

The consistently higher BP readings from PTT compared to ABPM were not unexpected. As hypothesized, the continuous nature of PTT-based monitoring likely detects transient BP surges during respiratory events in sleep [10]. This notion is supported by the more pronounced discrepancy observed in severe OSA than in mild OSA, suggesting that frequent and intense respiratory disturbances in severe OSA lead to larger BP fluctuations, which are captured by PTT. This may help explain the observations of Nyvad et al. and Krisai et al., who noted greater discrepancies between nighttime PTT- and ABPM-derived BP than those observed in daytime measurements [14, 15].

Our findings showed that PTT-derived SBP, but not PTT-derived DBP, was significantly elevated in patients with severe OSA. However, some studies proposed that elevated DBP might be more closely linked to physiological responses triggered by respiratory disturbances during sleep [20, 21] The discrepancy might be explained by at least two reasons. First, the difference in each cohort’s demographics, factors such as hypertensive status, age, sex and vascular stiffness can influence the magnitude of this association [21–23]. For example, increased vascular stiffness often elevates SBP while potentially lowering DBP [24]. Second, regarding the PTT technique itself, PTT measurement depends on PWV, and it is also sensitive to multiple factors, including anthropometric variables, age-related vascular stiffness, and cardiac variability during apneic events [25]. Consequently, the exact PTT-derived value may be less reliable when these factors are unaccounted for or deviate from normative assumptions. Therefore, under the current algorithm, PTT-derived BP may be better suited for tracking trends rather than determining precise BP values.

Regarding the detection of nocturnal and masked hypertension, PTT-derived SBP showed reasonable sensitivity for both conditions, suggesting potential utility as a screening tool. The setup for PTT is relatively simple and is already integrated into certain home testing devices. Nonetheless, the threshold required modification; we identified the optimal cutoff for PTT-SBP for determining nocturnal hypertension was 104 mmHg. This relatively low cutoff could reflect the wide limit of agreement of two measurements, which may be attributed to the varying degree of OSA severity among participants. In our study, the discrepancy between PTT-BP and ABPM was more pronounced in patients with moderate and severe OSA compared to those with mild disease. This variability suggests that future prospective validation should be stratified by OSA severity. In addition, the clinical utility of this approach might not be appropriate for patients with cardiac diseases [26],

In our study, DBP provided less predictive value for both nocturnal and masked hypertension. This finding might explain the poorer diagnostic performance of PTT when its criteria include DBP. When hypertension was defined by SBP alone (SBP > 120 mmHg), PTT demonstrated superior accuracy relative to diastolic-based criteria. In one study, the correlation between PTT-derived DBP and ABPM-DBP was weaker than the correlation for SBP [16]. Physiologic investigations propose that DBP measurement by PTT needs additional correction for the pre-ejection period, which is the interval between electrical cardiac activation and mechanical ventricular ejection, further complicating its assessment [27]. These factors highlight the inherent limitations in relying on PTT to yield exact BP values, especially diastolic pressure.

This study has several limitations. First, the PTT-derived BP and ABPM were not measured simultaneously, preventing the establishment of agreement limits between the two methods. This approach was chosen to avoid the interference of ABPM with PSG recordings and to better mirror real-world clinical conditions, which ABPM and PSG are usually not performed at the same time. The weak correlation should be interpreted with caution, as it could be attributed to both night-to-night variability and disagreement between the measurement methods. Nonetheless, ABPM was conducted on the following day, making significant changes in hypertensive status unlikely within that short interval. Second, the exclusion of patients receiving split-night CPAP titration, intended to avoid confounding from CPAP-induced BP alterations, produced a cohort with distinctive demographics (more females and less severe OSA). This demographic shift may limit the applicability of our PTT-derived BP results to full-night PSG studies and a narrower range of OSA severity. Future research may examine whether averaging PTT-derived BP values from the first half of the night, before CPAP initiation, yields comparable and clinically meaningful outcomes. Third, there are various physiological factors affecting systolic and diastolic pressures that appear to affect the correlation between PTT- and ABPM-based that have not been measured. Advanced arterial stiffness in some patients with OSA may be a confounder limiting the accuracy of PTT. Further investigations focusing on specific age groups and measuring vascular stiffness might clarify this relationship. Additionally, capturing BP fluctuation, instead of exact value, using PTT may be feasible. This is suggested by a study in which very short-term PTT-derived BP variation was linked to adverse events in patients with heart failure [28]. Fourth, while the relatively small sample size is sufficient for primary analyses, it limits the power of subgroup analyses and the generalizability of the derived diagnostic cutoffs. A larger, external validation cohort is required. Finally, we could not determine the clinical significance of elevated PTT-derived BP in the absence of elevated ABPM results. It is of great interest whether these patients carry a higher risk of developing clinical hypertension. PTT could be useful for the early detection of patients who are at risk and providing a window for timely intervention. Prospective studies linking cardiovascular outcomes to nocturnal or masked hypertension detected by PTT are needed to delineate the utility of PTT-derived BP as a screening or adjunctive monitoring method.

## Conclusion

We observed that PTT-derived BP was significantly higher than ABPM readings in patients with moderate to severe OSA. Although multiple factors may complicate the precise interpretation of these PTT-based measurements, PTT-SBP shows promise as a screening modality for identifying nocturnal and masked hypertension. Additional investigations should address the influence of patient characteristics to PTT value, especially arterial stiffness. The distinct properties of SBP and DBP also warrant caution when interpreting nocturnal BP elevations measured by PTT in OSA.

## Supplementary Information

Below is the link to the electronic supplementary material.

Supplementary file1 (DOCX 69.6 KB)

## Data Availability

The data that support the findings of this study are available from the corresponding author upon reasonable request.

## Declarations

All authors have read and agreed with the version of the submitted manuscript. The abstract of this manuscript was presented as a poster at the APSS annual meeting in Seattle, WA, USA from June 8–11, 2025.

This study was approved and overseen by the Institutional Review Board of the Faculty of Medicine Siriraj Hospital, Mahidol University (Si 482/2023).

## Abbreviations

ABPM: Ambulatory Blood Pressure Monitoring
AHI: Apnea–Hypopnea Index
AUC: Area Under the Curve
BP: Blood Pressure
DBP: Diastolic Blood Pressure
ECG: Electrocardiogram
EEG: Electroencephalogram
EMG: Electromyography
EOG: Electrooculography
ISO: International Organization for Standardization
IQR: Interquartile Range
ODI: Oxygen Desaturation Index
OSA: Obstructive Sleep Apnea
PLMI: Periodic–Limb–Movement Index
PSG: Polysomnography
PTT: Pulse Transit Time
PWV: Pulse Wave Velocity
ROC: Receiver Operating Characteristic
SBP: Systolic Blood Pressure
SpO₂: Peripheral Oxygen Saturation
USA: United States of America
WASO: Wakefulness After Sleep Onset
