# Supplementary material for: Comparative assessment of pulse transit time–derived blood pressure and ambulatory blood pressure monitoring in patients with obstructive sleep apnea
Source: Sleep Breath. 2026 Feb 11;30(1):38. doi: 10.1007/s11325-026-03601-6 (PMC12894130; doi:10.1007/s11325-026-03601-6)
Supplement: Supplementary file 1 — Supplementary file1 (DOCX 69.6 KB) [file 11325_2026_3601_MOESM1_ESM.docx]

**SUPPLEMENTARY MATERIALS**

**FIGURE 3** Receiver operating characteristic curve for pulse transit time–derived systolic blood pressure detecting *nocturnal hypertension*.
The curve plots sensitivity versus 1 – specificity for pulse transit time–derived systolic blood pressure in predicting a mean nighttime blood pressure ≥ 120/70 mm Hg (ambulatory blood pressure monitoring reference). The area under the curve is 0.62 (95% CI 0.48–0.77). Coordinates and Youden index values (optimum threshold approximately 104 mm Hg) are provided in the accompanying table.


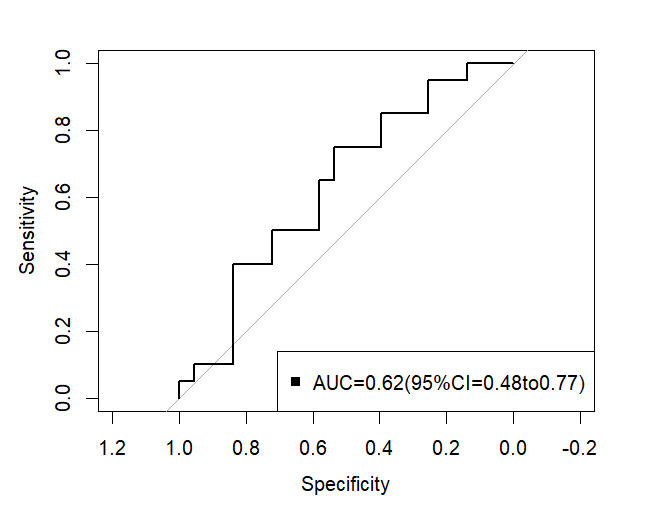


| **Threshold (mmHg)** | **Specificity** | **Sensitivity** | **Youden’s J Index** |
| --- | --- | --- | --- |
| -Inf | 0.00000000 | 1.00 | 0.00000000 |
| 87.5 | 0.02325581 | 1.00 | 0.02325581 |
| 92.0 | 0.06976744 | 1.00 | 0.06976744 |
| 93.5 | 0.11627907 | 1.00 | 0.11627907 |
| 95.0 | 0.13953488 | 0.95 | 0.08953488 |
| 96.5 | 0.16279070 | 0.95 | 0.11279070 |
| 97.5 | 0.23255814 | 0.95 | 0.18255814 |
| 98.5 | 0.25581395 | 0.95 | 0.20581395 |
| 99.5 | 0.25581395 | 0.90 | 0.15581395 |
| 101.0 | 0.25581395 | 0.85 | 0.10581395 |
| 102.5 | 0.32558140 | 0.85 | 0.17558140 |
| **104.0** | **0.39534884** | **0.85** | **0.24534884** |
| 105.5 | 0.39534884 | 0.80 | 0.19534884 |
| 106.5 | 0.39534884 | 0.75 | 0.14534884 |
| 107.5 | 0.44186047 | 0.75 | 0.19186047 |
| 110.5 | 0.53488372 | 0.70 | 0.23488372 |
| 113.5 | 0.53488372 | 0.65 | 0.18488372 |
| 114.5 | 0.58139535 | 0.60 | 0.18139535 |
| 115.5 | 0.58139535 | 0.50 | 0.08139535 |
| 116.5 | 0.72093023 | 0.40 | 0.12093023 |
| 117.5 | 0.74418605 | 0.40 | 0.14418605 |
| 119.0 | 0.76744186 | 0.40 | 0.16744186 |
| 120.5 | 0.79069767 | 0.40 | 0.19069767 |
| 121.5 | 0.81395349 | 0.40 | 0.21395349 |
| 122.5 | 0.83720930 | 0.35 | 0.18720930 |
| 123.5 | 0.83720930 | 0.30 | 0.13720930 |
| 126.0 | 0.83720930 | 0.25 | 0.08720930 |
| 128.5 | 0.83720930 | 0.20 | 0.03720930 |
| 130.0 | 0.83720930 | 0.10 | -0.06279070 |
| 132.5 | 0.90697674 | 0.10 | 0.00697674 |
| 135.0 | 0.93023256 | 0.10 | 0.03023256 |
| 136.5 | 0.95348837 | 0.10 | 0.05348837 |
| 138.5 | 0.95348837 | 0.05 | 0.00348837 |
| 141.5 | 1.00000000 | 0.05 | 0.05000000 |
| Inf | 1.00000000 | 0.00 | 0.00000000 |

| **FIGURE 4** Receiver operating characteristic curve for pulse transit time–derived diastolic blood pressure detecting *nocturnal hypertension*. This curve evaluates pulse transit time–derived diastolic blood pressure against the same nocturnal-hypertension standard as in Figure A (a mean nighttime blood pressure ≥ 120/70 mm Hg, as determined by ambulatory blood pressure monitoring). The area under the curve is 0.58 (95% CI 0.42–0.74). The table lists all thresholds, sensitivities, specificities, and Youden index values (optimum threshold approximately 78.5 mm Hg).  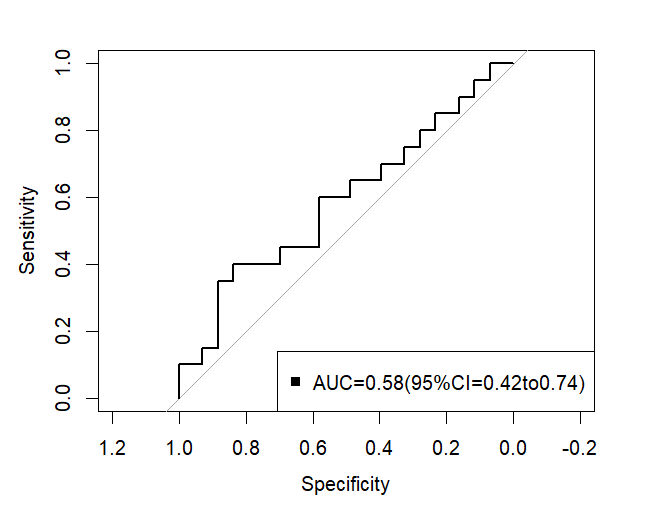 |
| --- |

| **Threshold (mmHg)** | **Specificity** | **Sensitivity** | **Youden’s J Index** |
| --- | --- | --- | --- |
| -Inf | 0.00000000 | 1.00 | 0.00000000 |
| 50.0 | 0.02325581 | 1.00 | 0.02325581 |
| 53.5 | 0.04651163 | 1.00 | 0.04651163 |
| 54.5 | 0.06976744 | 1.00 | 0.06976744 |
| 55.5 | 0.06976744 | 0.95 | 0.01976744 |
| 57.5 | 0.09302326 | 0.95 | 0.04302326 |
| 59.5 | 0.11627907 | 0.90 | 0.01627907 |
| 60.5 | 0.16279070 | 0.90 | 0.06279070 |
| 61.5 | 0.16279070 | 0.85 | 0.01279070 |
| 62.5 | 0.20930233 | 0.85 | 0.05930233 |
| 63.5 | 0.23255814 | 0.85 | 0.08255814 |
| 64.5 | 0.23255814 | 0.80 | 0.03255814 |
| 65.5 | 0.27906977 | 0.75 | 0.02906977 |
| 66.5 | 0.30232558 | 0.75 | 0.05232558 |
| 67.5 | 0.32558140 | 0.70 | 0.02558140 |
| 69.0 | 0.39534884 | 0.65 | 0.04534884 |
| 70.5 | 0.48837209 | 0.65 | 0.13837209 |
| 71.5 | 0.48837209 | 0.60 | 0.08837209 |
| 72.5 | 0.58139535 | 0.55 | 0.13139535 |
| 73.5 | 0.58139535 | 0.45 | 0.03139535 |
| 74.5 | 0.65116279 | 0.45 | 0.10116279 |
| 75.5 | 0.67441860 | 0.45 | 0.12441860 |
| 76.5 | 0.69767442 | 0.40 | 0.09767442 |
| 77.5 | 0.74418605 | 0.40 | 0.14418605 |
| **78.5** | **0.81395349** | **0.40** | **0.21395349** |
| 79.5 | 0.83720930 | 0.35 | 0.18720930 |
| 80.5 | 0.88372093 | 0.30 | 0.18372093 |
| 82.0 | 0.88372093 | 0.20 | 0.08372093 |
| 84.0 | 0.88372093 | 0.15 | 0.03372093 |
| 85.5 | 0.93023256 | 0.10 | 0.03023256 |
| 88.0 | 0.95348837 | 0.10 | 0.05348837 |
| 90.5 | 0.97674419 | 0.10 | 0.07674419 |
| 96.5 | 1.00000000 | 0.10 | 0.10000000 |
| 102.5 | 1.00000000 | 0.05 | 0.05000000 |
| Inf | 1.00000000 | 0.00 | 0.00000000 |

**FIGURE 5** Receiver operating characteristic curve for pulse transit time–derived systolic blood pressure detecting *masked hypertension*.
The curve assesses pulse transit time–derived systolic blood pressure performance in identifying masked hypertension, defined by abnormal daytime or nighttime ambulatory blood pressure monitoring in patients without a prior hypertension diagnosis. The area under the curve is 0.58 (95% CI 0.38–0.77). Threshold coordinates and Youden indices (optimum threshold approximately 104 mm Hg) accompany the plot.


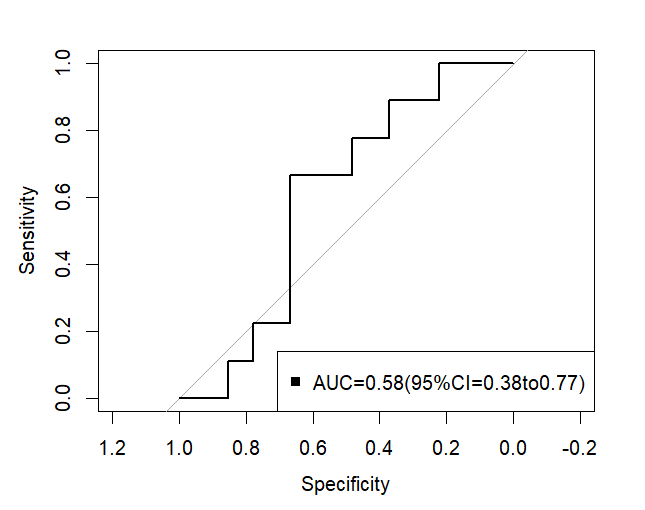


| **Threshold (mmHg)** | **Specificity** | **Sensitivity** | **Youden’s J Index** |
| --- | --- | --- | --- |
| -Inf | 0.00000000 | 1.0000000 | 0.00000000 |
| 87.5 | 0.03703704 | 1.0000000 | 0.03703704 |
| 92.0 | 0.11111111 | 1.0000000 | 0.11111111 |
| 93.5 | 0.18518519 | 1.0000000 | 0.18518519 |
| 95.0 | 0.22222222 | 0.8888889 | 0.11111111 |
| 96.5 | 0.25925926 | 0.8888889 | 0.14814815 |
| 97.5 | 0.33333333 | 0.8888889 | 0.22222222 |
| **99.0** | **0.37037037** | **0.8888889** | **0.25925926** |
| 101.0 | 0.37037037 | 0.7777778 | 0.14814815 |
| 102.5 | 0.44444444 | 0.7777778 | 0.22222222 |
| **104.5** | **0.48148148** | **0.7777778** | **0.25925926** |
| 106.5 | 0.48148148 | 0.6666667 | 0.14814815 |
| 107.5 | 0.55555556 | 0.6666667 | 0.22222223 |
| 110.5 | 0.66666667 | 0.4444444 | 0.11111111 |
| 114.0 | 0.66666667 | 0.3333333 | 0.00000000 |
| 115.5 | 0.66666667 | 0.2222222 | -0.11111111 |
| 118.5 | 0.77777778 | 0.1111111 | -0.11111111 |
| 121.5 | 0.81481481 | 0.1111111 | -0.07407407 |
| 123.0 | 0.85185185 | 0.1111111 | -0.03703704 |
| 127.5 | 0.85185185 | 0.0000000 | -0.14814815 |
| 132.5 | 0.92592593 | 0.0000000 | -0.07407407 |
| 135.0 | 0.96296296 | 0.0000000 | -0.03703704 |
| Inf | 1.00000000 | 0.0000000 | 0.00000000 |

**FIGURE 6** Receiver operating characteristic curve for pulse transit time–derived diastolic blood pressure detecting *masked hypertension*.
This curve presents pulse transit time–derived diastolic blood pressure accuracy for masked hypertension; the area under the curve is 0.51 (95% CI 0.29–0.73). A full table of thresholds, sensitivities, specificities, and Youden indices (optimum threshold approximately 64 mm Hg) accompany the plot.


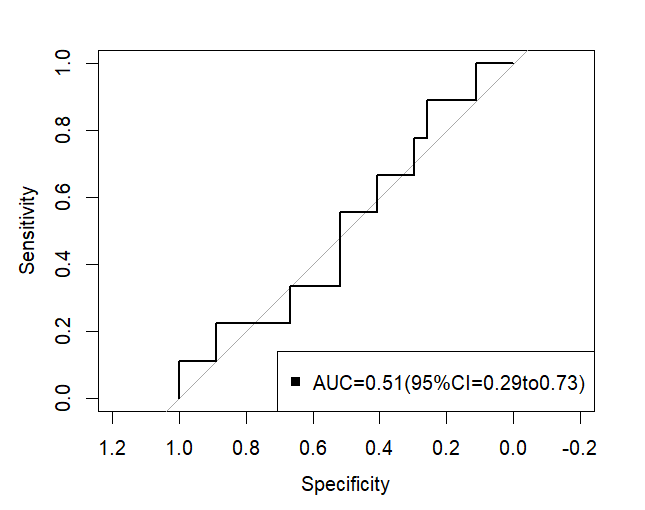


| **Threshold (mmHg)** | **Specificity** | **Sensitivity** | **Youden’s J Index** |
| --- | --- | --- | --- |
| -Inf | 0.00000000 | 1.0000000 | 0.00000000 |
| 50.0 | 0.03703704 | 1.0000000 | 0.03703704 |
| 53.5 | 0.07407407 | 1.0000000 | 0.07407407 |
| 54.5 | 0.11111111 | 1.0000000 | 0.11111111 |
| 55.5 | 0.11111111 | 0.8888889 | 0.00000000 |
| 57.5 | 0.14814815 | 0.8888889 | 0.03703704 |
| 60.5 | 0.18518519 | 0.8888889 | 0.07407407 |
| 62.5 | 0.22222222 | 0.8888889 | 0.11111111 |
| **64.0** | **0.25925926** | **0.8888889** | **0.14814815** |
| 65.5 | 0.25925926 | 0.7777778 | 0.03703704 |
| 66.5 | 0.29629630 | 0.7777778 | 0.07407407 |
| 67.5 | 0.29629630 | 0.6666667 | -0.03703703 |
| 69.0 | 0.33333333 | 0.6666667 | 0.00000000 |
| 70.5 | 0.40740741 | 0.6666667 | 0.07407407 |
| 71.5 | 0.40740741 | 0.5555556 | -0.03703703 |
| 72.5 | 0.51851852 | 0.4444444 | -0.03703704 |
| 73.5 | 0.51851852 | 0.3333333 | -0.14814815 |
| 74.5 | 0.59259259 | 0.3333333 | -0.07407407 |
| 75.5 | 0.62962963 | 0.3333333 | -0.03703704 |
| 76.5 | 0.66666667 | 0.2222222 | -0.11111111 |
| 77.5 | 0.74074074 | 0.2222222 | -0.03703704 |
| 79.0 | 0.85185185 | 0.2222222 | 0.07407407 |
| 80.5 | 0.88888889 | 0.2222222 | 0.11111111 |
| 83.0 | 0.88888889 | 0.1111111 | 0.00000000 |
| 87.5 | 0.96296296 | 0.1111111 | 0.07407407 |
| 96.5 | 1.00000000 | 0.1111111 | 0.11111111 |
| Inf | 1.00000000 | 0.0000000 | 0.00000000 |
